# Mutant Prevention Concentration of Ciprofloxacin against *Klebsiella pneumoniae* Clinical Isolates: An Ideal Prognosticator in Treating Multidrug-Resistant Strains

**DOI:** 10.1155/2019/6850108

**Published:** 2019-10-20

**Authors:** B. Aditi Priyadarshini, Krishnan Mahalakshmi, Venkatesan Naveen Kumar

**Affiliations:** ^1^Department of Microbiology, Bharath Institute of Higher Education and Research, Velachery, Tambaram Road, Chennai 600100, Tamilnadu, India; ^2^Research Lab for Oral-Systemic Health Sree Balaji Dental College and Hospital, Bharath Institute of Higher Education and Research, Velachery, Tambaram Road, Chennai 600100, Tamilnadu, India; ^3^ImmuGenix Biosciences Pvt Ltd., Chennai, Tamilnadu, India

## Abstract

**Background:**

Fluoroquinolone-resistant* Klebsiella pneumoniae* poses a therapeutic challenge when implicated in urinary tract infections, pyelonephritis, pneumonia, skin infections, osteomyelitis, and respiratory infections. The mutant prevention concentration (MPC) represents a concentration threshold above which increase of resistant mutants occurs rarely. The aim of the present study is to determine the MPC among ciprofloxacin-resistant *K. pneumoniae* clinical isolates.

**Materials and Methods:**

A total of 240 clinical isolates of *K. pneumoniae* were collected from a tertiary care hospital. The MPCs were determined for 24 selected strains using an inoculum of 10^10^ CFU/ml in Müller–Hinton agar plates with serial/various concentrations (0.003–100 *μ*g) of ciprofloxacin. In addition to the MPC, phenotypic screening for ESBL, AmpC, and carbapenemase was performed. The detection of *qnr* genes for 24 isolates and DNA sequencing for six isolates were performed.

**Results:**

Ciprofloxacin resistance was observed in 19.6% of the *K. pneumoniae* clinical isolates. Among the ciprofloxacin-resistant isolates, 14 isolates showed an MPC value of more than 100 *μ*g. The MPC ranged between 100 *μ*g and 20 *μ*g for ciprofloxacin-resistant isolates. ESBL producers and *qnr* gene-producing strains had a high MPC. 11 isolates showed the presence of either *qnrB* or *qnrS* genes. None of the samples showed the presence of the *qnrA* gene.

**Conclusion:**

From our study, we infer that ESBL producers and *qnr* gene-possessing strains are frequently resistant to ciprofloxacin. Estimation of the MPC in the case of multidrug-resistant isolates in the clinical setup may help in treating these drug-resistant strains.

## 1. Introduction

Fluoroquinolones have phenomenal clinical effect against *Enterobacteriaceae* including *Klebsiella*. However, the recurrence of ciprofloxacin-resistant *Klebsiella pneumoniae* has expanded worldwide lately [1, 2]. The oral bioavailability of these agents is good such that tissue and fluid concentrations often exceed the serum drug concentration [3–5]. Antibiotic resistance among human pathogens is currently on the rise in every bacterial species for which an alternative modality of antibiotic therapy is warranted [6–8]. The original fluoroquinolone agents were introduced in the late 1980s. Shortly thereafter, ciprofloxacin became the most frequently used antibiotic throughout the world [9]. Ciprofloxacin is the most potent second-generation fluoroquinolone which has broader clinical applications in the treatment of complicated urinary tract infections, pyelonephritis, sexually transmitted diseases, pneumonia, skin infections, osteomyelitis, and respiratory infections [10–12].

The mechanism by which quinolones affect bacterial DNA synthesis is by inhibiting DNA gyrase and type IV topoisomerase in Gram-negative bacteria and Gram-positive bacteria, respectively. Both DNA gyrase and type IV topoisomerase are essential for relaxing DNA supercoils during DNA replication [13–15].

The resistance of Gram-negative bacteria to fluoroquinolones is reported concomitant with their use [16–18]. Bacterial mutations occur during quinolone therapy, thereby limiting its usefulness in containing infections. This resistance can occur by any three mechanisms, namely, alterations in quinolone enzymatic targets (DNA gyrase), decreased outer membrane permeability, and the development of efflux mechanisms.

The development of very high minimum inhibitory concentrations to ciprofloxacin in isolates of *Enterobacteriaceae* species, *Staphylococcus aureus*, and *P. aeruginosa* [19] is due to the result of accumulation of several bacterial mutants. Alterations in bacterial permeability and the development of efflux pumps are the other mechanisms of resistance. This resistance mechanism is shared with resistance to other antimicrobial agents structurally unrelated to quinolones, such as beta lactams, tetracyclines, and chloramphenicol [20]. Such a resistance to different classes of antibiotics has increased in the recent years and is especially high in extended-spectrum beta lactamase- (ESBL-) producing strains [21, 22].

Although chromosomal mutations commonly result in quinolone resistance, plasmid-mediated resistance has been discovered recently. The gene responsible for plasmid-mediated resistance, *qnr*, was found on plasmids varying in size from 54–≥180 kb in clinical isolates of *K. pneumoniae* and *Escherichia coli* from which low-level quinolone resistance could be transferred to a sensitive recipient by conjugation [23, 24]. Plasmid-mediated quinolone resistance is mediated by the genes (*qnr*) encoding for proteins that belong to the pentapeptide repeat family and protect DNA gyrase and topoisomerase IV against quinolone compounds [25]. The three major groups of *qnr* determinants are *qnrA*, *qnrB*, and *qnrS* [26, 27].

The mutant prevention concentration (MPC) is a novel concept coined by Dong and colleagues following the recognition of a two-stage decline in colony forming units when high-density bacterial inocula were exposed to varying antimicrobial drug concentrations [28].

Dong et al. have defined the mutant prevention concentration (MPC) as a parameter which prevents or interferes with the emergence and development of resistant mutants. Such resistant mutants may be associated with treatment failures and surfacing of resistance [28]. MPC represents a concentration threshhold above which increase of resistant mutants occurs rarely [29]. It appeared that the MPC might serve as a simple measure of antibiotic potency that incorporates the ability of a compound to restrict a selection of resistant mutants [30]. The mutant selection window (MSW) defines the range of drug concentration in which resistant mutants develop easily. The upper limit of the MSW is the MPC value and the lower limit is the MIC value [29].

Currently, the drug resistance among Gram-negative bacilli is on the rise with regard to ESBL-producing *K. pneumoniae*. Recently, plasmid-mediated resistance to quinolones was identified for the first time in clinical strains of *K. pneumoniae* [23]. Quinolone resistance is found with a surprisingly high frequency (18% to 56%) in extended-spectrum *β*-lactamase- (ESBL-) producing isolates [31–33]. Fluoroquinolones may be given in higher doses to prevent the selection of mutant populations, especially in ESBL-positive strains, by administering higher concentrations (above the MPC) of fluoroquinolones at the site of infection.

Hence, the aim of the present study is to determine the MPC among ciprofloxacin-resistant *K. pneumoniae* clinical isolates.

## 2. Materials and Methods

### 2.1. Bacterial Isolates

A total of 240 clinical isolates of *K. pneumoniae* were collected from a tertiary care hospital between October 2015 and December 2018. The source of the clinical isolates included blood (*n*=7), pus (*n*=8), sputum (*n*=10), urine (*n*=170), vaginal swab (*n*=28), and wound swab (*n*=7). Out of the 240 isolates, 47 were resistant to ciprofloxacin. The ciprofloxacin-resistant (*n*=20) and ciprofloxacin-susceptible (*n*=4) isolates were subjected to an MPC assay.

To consider the strains positive for ESBL, a modified CLSI-ESBL confirmatory test [34] was performed employing disks of cefotaxime (CTX) and ceftazidime (CTZ), on which both boronic acid (BA) and ethylene diamine tetra acetic acid (EDTA) were dispensed. The agar plates were incubated at 37°C for 18 h. An augmentation of ≥5 mm in the growth inhibitory zone diameter of either CTX-CA or CAZ-CA in combination with BA and EDTA compared with the zone diameter of CTX or CAZ disks containing BA and EDTA was considered positive for ESBL production. For detecting carbapenemase, disks of MER (10 *μ*g) alone and with 400 *μ*g of phenyl boronic acid and 292 *μ*g of EDTA were used [35]. Phenotypic detection of AmpC production was carried out by using disks of cefotetan with and without BA [36].

### 2.2. Determination of the MIC and MPC

The ciprofloxacin MIC was determined for all clinical isolates using VITEK 2 systems version 7.0 as per CLSI guidelines. MPC was analyzed for 24 clinical isolates as per the study of Blondeau et al. [37]. Fresh cultures of bacterial isolates grown on Müller–Hinton agar (MHA) incubated overnight at 37°C were included for the MPC assay. A bacterial suspension of ≥3 × 10^10^ CFU/ml was prepared in peptone water and compared with McFarland's standard 1. For MPC determination, 100 *μ*l of the bacterial suspension was inoculated onto MHA plates containing ciprofloxacin. The concentration of ciprofloxacin ranged between 200 and 0.003 *μ*g. These plates were incubated at 37°C and observed for colonies every 24 hours over 2 days to confirm the stabilization of the colony number.

### 2.3. Screening and Sequencing of *qnr* Genes

DNA was extracted by the boiling lysis method. The supernatant was transferred to a sterile tube and stored at 20°C until assaying. Conventional PCR was used to detect the presence of *qnrA*, *qnrB*, and *qnrS* genes. The PCR master mix (25 *μ*l) contained sterile Milli Q water (18.4 *μ*l), 10x PCR buffer (2.5 *μ*l), dNTP mix (1 *μ*l), 16S forward primer (1 *μ*l), 16S reverse primer (1 *μ*l), *Taq* polymerase enzyme (0.1 *μ*l), and DNA template (1 *μ*l). The primers used for screening *qnr* genes are mentioned in Table 1 [38]. The thermal cycling conditions during the initial denaturation were 95°C for 5 min, followed by 30 cycles of denaturation at 95°C for 45 seconds. Annealing was performed at 55°C for 30 seconds for *qnrA* and 60°C for *qnrB* and *qnrS* and extended for 45 seconds at 72°C. The final extension was at 72°C for 5 minutes. The amplified products were detected by electrophoresis on 1% agarose gel with ethidium bromide and visualized under a UV transilluminator.

### 2.4. Gene Sequencing

PCR amplicons of the *qnr* genes were sequenced using the specific primer as described above. Sequencing was carried out at Macrogen Inc. (Seoul, Korea) using an ABI PRISM® BigDye™ Terminator and ABI 3730XL sequencer (Applied Biosystem, USA). The *qnr* gene sequences of representative isolates were compared with known sequences in the NCBI Database by using BLAST analysis (http://www.ncbi.nlm.nih.gov/blast/). The sequences were submitted to GenBank under accession numbers MK 414320, MK414318, MK414319, MK414320, MK414321, and MK414322.

## 3. Results

### 3.1. Determination of the MIC and MPC

Among the 240 clinical isolates of *K. pneumoniae*, 47 isolates showed resistance to ciprofloxacin. The source of the resistant isolates were blood (*n*=2), Pus (*n*=1), sputum (*n*=3), urine (*n*=31), vaginal swab (*n*=6), and wound swab (*n*=4). All ciprofloxacin-resistant isolates showed similar MIC values (≥4). There were nine multidrug-resistant strains. None of the isolates were resistant to the last-resort drug colistin.

**Figure 1:**
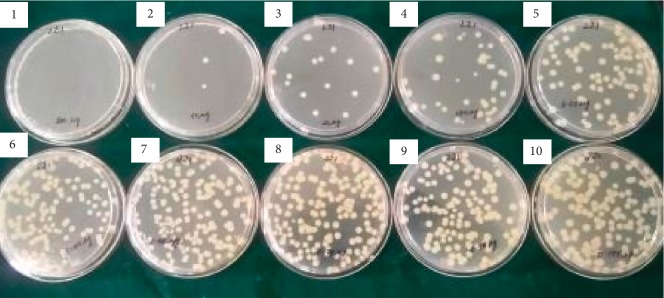
A representative image showing an MPC of 100 *μ*g against MDR *Klebsiella pneumoniae*. Complete growth inhibition at 100 *μ*g concentration of ciprofloxacin.

**Figure 2:**
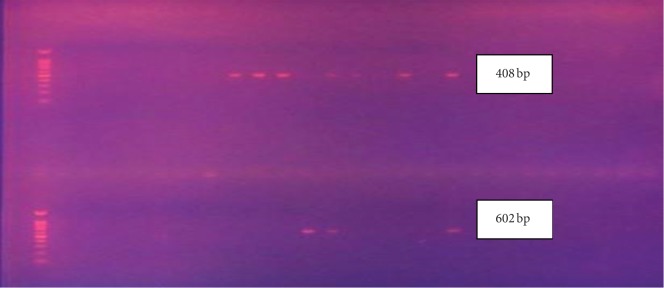
A representative gel image showing PCR products of *qnrB* (408 bp) and *qnrS* (602 bp).

The MIC and MPC results of ciprofloxacin for the 24 isolates are shown in Table 2. The MIC value for ciprofloxacin-susceptible strains was ≤1, while the MIC of the resistant strains was ≥4, irrespective of the strains being positive for ESBL, AmpC, or carbapenemases. Clinical isolates of *K. pneumoniae* which showed resistance to ciprofloxacin (*n*=47) were randomly chosen (*n*=20) for the determination of the MPC. Four susceptible isolates were used as the control. Among the ciprofloxacin-resistant isolates, 14 isolates showed an MPC value of more than 100 *μ*g (Figure 1). Rest of the isolates showed an MPC value of 100 *μ*g. The MPC ranged between 100 *μ*g and 200 *μ*g among the ciprofloxacin-resistant clinical isolates of *K. pneumoniae*. The MPC range for ciprofloxacin-susceptible strains was between 3.15 *μ*g and 50 *μ*g. Two isolates showed high MPC values of 175 *μ*g and 200 *μ*g, and these two isolates were phenotypically positive for AmpC, ESBL, and carbapenemase and genotypically positive for *qnrB*. 11 of these 24 isolates were found to possess either *qnrB* (*n*=8) or *qnrS* (*n*=4) genes. Only one isolate was positive for both *qnrB* and *qnrS* genes (Figure 2). The *qnrA* gene was not detected in any of the 24 isolates (Table 2). The ciprofloxacin-resistant isolates (*n*=20) which were phenotypically positive for ESBL (*n*=6), AmpC (*n*=8), and carbapenemases (*n*=5) showed the presence of either *qnrB* or *qnrS* genes.

## 4. Discussion

*Klebsiella pneumoniae* strains producing ESBL and AmpC are a major health problem because of the difficulty in treating severe infections caused by these microorganisms. Furthermore, they may show resistance to other groups of antimicrobial agents [32]. As these strains frequently cause urinary tract infections, cross resistance with fluoroquinolones is a very challenging issue since intravenous therapy is not recommended in these infections except for the most serious one (pyelonephritis). Fluoroquinolone resistance among the ESBL strains is an added burden in treating patients with complicated UTI [29]. In our study, 11 out of 20 ciprofloxacin-resistant isolates were also ESBL producers. This finding is in line with Tolun et al. [39], while this result is not in concurrence with Azargun et al., who showed 34% ESBL production [40]. Fluoroquinolones are preferred for treating complicated UTI and prostatitis as they have a high renal concentration and Gram-negative antibacterial activity. One of the significant limiting factors in using fluoroquinolones is the development of mutations during treatment [14]. Daoud et al. have reported the difficulty in treating ciprofloxacin-susceptible ESBL producers as their MPC is high. The results of our study among ciprofloxacin-resistant isolates with ESBL production showed a high MPC, which is in concurrence with the study of Daoud et al.[29]. The plasmid-mediated *qnr* genes were detected with high frequency among ciprofloxacin-resistant *Klebsiella* isolates and these strains also exhibited a high MIC and MPC. Some of the ciprofloxacin-resistant isolates which did not show the presence of *qnr* genes also showed an elevated MPC. Such an elevated MPC among isolates that were positive for *qnr* may lead to resistant mutants. This was well in line with our results shown by resistant strains of *K. pneumoniae* [41]. Studies with larger bacterial isolates may clarify the bidirectional relationship between the presence of *qnr* genes and a high MPC among resistant isolates. The strains which did not show the presence of any of the genes might harbour other variants of *qnr* genes. Treatment with higher doses of fluoroquinolones above the MPC value in the case of multidrug-resistant strains can be provided to eradicate these isolates at the site of infection. The MPC values may be considered to plan the dosage for treating patients infected with isolates showing coresistance to ciprofloxacin and ESBL [7, 42]. The ciprofloxacin bioavailability of different dosages at different body sites may be an added assistance in the treatment plan of MDR. The bioavailability of ciprofloxacin at different body sites for oral and intravenous routes has been reported by Brunner et al. [43].

The concept of the MPC can be considered to make therapeutic decisions about the choice and dosage of antibiotics to control the development of resistant mutants in the *Enterobacteriaceae* family. Our study also emphasizes the frequent finding of resistance to fluoroquinolones in ESBL-producing *K. pneumoniae* strains. The determination of MPCs in the case of multidrug-resistant isolates in the clinical setup may be significant in therapeutic planning.

## Figures and Tables

**Table 1:** Primers used for screening of *qnr* genes in *K. pneumoniae* clinical isolates.

Gene	Primers
*qnrA*	F-5′-TTCAGCAAGAGGATTTCTCA-3′
	R-5′-GGCAGCACTATTACTCCCAA-3′
*qnrB*	F-5′-CCTAGCGGCACTGAATTTAT-3′
	R-5′-GTTTGCTGCTCGCCAGTCGA-3′
*qnrS*	F-5′-CAATCATACATATCGGCACC-3′
	R-5′-TCAGGATAAACAACAATACCC-3′

**Table 2:** MPC, MIC, ESBL, AmpC, and carbapenemase detection in clinical isolates of *K. pneumoniae*.

S no.	Isolate no.	Source	MIC′	MPC′	Genes	ESBL	AmpC	Carbapenemases
1	28 (S)	Urine	≤0.25	25	—	+	−	−
2	56 (S)	Urine	≤0.25	50	—	−	−	−
3	126 (S)	Urine	1	25	—	+	+	+
4	198 (S)	Urine	≤0.25	3.125	—	−	−	−
5	210^*∗*^	Urine	≥4	175	—	−	−	+
6	211^*∗*^	Urine	≥4	100	—	+	−	+
7	212^*∗*^	Urine	≥4	12.5	—	−	+	+
8	213^*∗*^	Urine	≥4	125	*qnrB*	−	−	−
9	215	Urine	≥4	125	*qnrB*	−	+	+
10	217^*∗*^	Urine	≥4	175	*qnrB*	+	+	+
11	218^*∗*^	Urine	≥4	125	*qnrS*	−	+	+
12	219^*∗*^	Sputum	≥4	125	*qnrB qnrS*	−	+	+
13	220^*∗*^	Sputum	≥4	200	*qnrB*	+	+	+
14	221	Urine	≥4	100	—	−	−	−
15	222^*∗*^	Sputum	≥4	125	*qnrB*	+	−	−
16	223	Urine	≥4	125	—	−	+	−
17	224	Vaginal swab	≥4	100	—	+	−	−
18	230	Urine	≥4	100	*qnrS*	+	+	−
19	231	Urine	≥4	125	—	+	+	−
20	232	Vaginal swab	≥4	125	*qnrB*	+	+	−
21	233	Urine	≥4	125	*qnrB*	−	+	−
22	236	Urine	≥4	125	*qnrS*	+	−	−
23	239	Urine	≥4	100	—	+	+	−
24	240	Urine	≥4	100	—	+	+	−

S, susceptible to ciprofloxacin; ^*∗*^, multidrug resistant; MIC′, minimum inhibitory concentration for ciprofloxacin in *μ*g; MPC′, mutant prevention concentration for ciprofloxacin in *μ*g.

## Data Availability

The data used to support the findings of this study are available from the corresponding author upon request.
